# Management of Endocrinopathies in Pregnancy: A Review of Current Evidence

**DOI:** 10.3390/ijerph16050781

**Published:** 2019-03-04

**Authors:** Daniela Calina, Anca Oana Docea, Kirill Sergeyevich Golokhvast, Stavros Sifakis, Aristides Tsatsakis, Antonis Makrigiannakis

**Affiliations:** 1 Department of Clinical Pharmacy, University of Medicine and Pharmacy of Craiova, 200349 Craiova, Romania; calinadaniela@gmail.com; 2 Department of Toxicology, University of Medicine and Pharmacy of Craiova, 200349 Craiova, Romania; 3 Scientific Education Center of Nanotechnology, Far Eastern Federal University, Vladivostok 690950, Russia; droopy@mail.ru; 4 Department of Obstetrics and Gynecology, Mitera Maternity Hospital, 71110 Heraklion, Crete, Greece; stavros.sifakis@yahoo.com; 5 Department of Forensic Sciences and Toxicology, Faculty of Medicine, University of Crete, 71110 Heraklion, Crete, Greece; tsatsaka@uoc.gr; 6 Department of Obstetrics and Gynecology, Medical School, University of Crete, 71110 Heraklion, Crete, Greece; makrygia@uoc.gr

**Keywords:** pregnancy, pharmacotherapeutic management, thyroid, parathyroid, adrenal disorders, pituitary, diabetes insipidus

## Abstract

Pregnancy in women with associated endocrine conditions is a therapeutic challenge for clinicians. These disorders may be common, such us thyroid disorders and diabetes, or rare, including adrenal and parathyroid disease and pituitary dysfunction. With the development of assisted reproductive techniques, the number of pregnancies with these conditions has increased. It is necessary to recognize symptoms and correct diagnosis for a proper pharmacotherapeutic management in order to avoid adverse side effects both in mother and fetus. This review summarizes the pharmacotherapy of these clinical situations in order to reduce maternal and fetal morbidity.

## 1. Introduction

Endocrine disorders and their treatment during pregnancy represent an important topic for clinicians, endocrinologists, obstetricians, gynecologists, and other medical specialties involved, due to their potential impact upon pregnancy and fetal development. Endocrine physiology for the mother and fetus is constantly changed across pregnancy and many endocrine events appear, partially explained by the development of the maternal–fetal unit in the placenta, a temporary gland. Both mother and fetus adapt using unique mechanisms, at this development during pregnancy, including changes of endocrine system in the mother and fetus, as well as related feedback changes. Initially, the endocrine function of the fetus is totally dependent on the mother because most of the endocrine glands start the hormonal production in the second trimester of pregnancy. Afterwards, the fetus is less dependent on the maternal endocrine function, but the fetal glands are continuously developing both in function and morphology until birth [1,2].

Clinical situations needing endocrine treatment during pregnancy are related to two different conditions: to continue treatment for a chronic endocrine disorder diagnosed before pregnancy or for the treatment of symptoms or disorders newly diagnosed during pregnancy. Drug treatment during pregnancy exposes the mother and fetus to adverse effects and potential risks, which depend on the gestational age. Clinical trials during pregnancy are rarely accepted, risky and expensive, requiring very strict ethical regulations and long-term follow-up [3].

In the first two weeks of pregnancy, the embryo is under “all-or-nothing” law, meaning that a drug could trigger embryonic death or it has no influence at all on the future development of pregnancy. In the next eight weeks until the end of the first trimester (the so-called major and minor organogenesis period) cellular differentiation and organogenesis occur. Any drug given during this period must be verified concerning the potential teratogenic risk and be free from causing congenital malformations. In the second and third trimester, the drugs could induce fetal toxicity, especially at the central nervous system level.

In order to classify their safety during pregnancy, the drugs were divided by Food and Drug Administration (FDA) in five categories [4].

1. Category A—Randomized control trials were not able to demonstrate any fetal risk in the first trimester of pregnancy, as well as no risk at all in the second and third trimester.
2. Category B—Studies on experimental animals were not able to demonstrate a fetal risk but there are no well-controlled adequate studies in pregnant women. Most of the drugs are included in this category.
3. Category C—Studies on experimental animals demonstrated an adverse effect upon fetus and there are no well-controlled adequate studies in pregnant women, but the potential benefits could justify the use of that drug in pregnant women, despite potential risks.
4. Category D—There are important proofs showing the human fetal risks, starting from data of adverse reactions on experimental animals, or human studies, but the potential benefits could justify the use of drug in pregnant women despite potential risks.
5. Category X—Studies on experimental animals and/or in humans from previously reported adverse effects or marketing studies demonstrate fetal anomalies and positive evidence on human-fetal risks. The risk of using the drug during pregnancy is far more than the benefits and women taking these drugs should avoid conception using contraceptive measures.

Women whose pregnancies are complicated by endocrine disorders have a major risk of developing maternal and fetal complications that can be minimized by appropriate management and clinical observation [5]. Medical literature has been reviewed in order to obtain data regarding the correct therapy of these clinical situations, some of these being very rare. 

This paper highlights the pharmacotherapeutic management of these conditions in order to avoid maternal and fetal complications during the gestational and postpartum period.

## 2. The Endocrine Disorders and Pregnancy

Most endocrinopathies associated with pregnancy can lead to maternal and fetal complications. In order to avoid them it is important to be treated properly. The initial diagnosis of many of them is often difficult due to the overlapping of symptoms occurring during normal pregnancy, such as hyperemesis gravidarum and those suggesting specific endocrine diseases, as well as changes in the baseline for common biochemical measurements resulting from physiological changes during pregnancy [5]. Hyperemesis gravidarum during pregnancy can be manifested by nausea, vomiting, weakness, drowsiness, poor health, irritability, and depression. It is caused by the increase of the human chorionic gonadotropin hormone (hCG) in the body of a pregnant woman. In the first trimester of pregnancy hCG stimulates the formation of hormones necessary for development and maintenance of pregnancy: progesterone and the estrogens estradiol and free estriol [5]. In the normal course of pregnancy, these hormones and human placental lactogen (hPL) will be secreted by the placenta. Placental lactogen hormone actively affects the metabolism, under its influence the intake of amino acids increases to “build” the baby’s tissues and cause nausea, headaches and fatigue to the mother [5].

### 2.1. Thyroid Disorders and Pregnancy

#### 2.1.1. Physiology of Thyroid in Pregnancy

**Figure 1:**
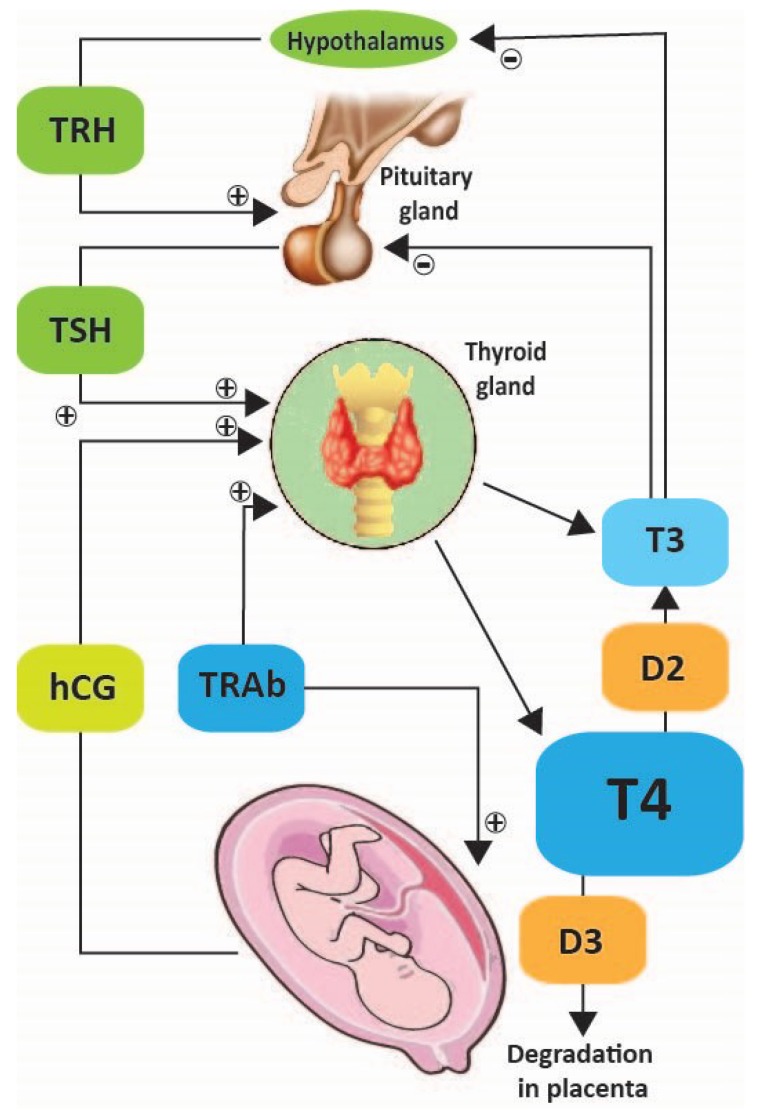
Pregnancy and hypothalamic pituitary thyroid axis. T4—Tetraiodothyronin, T3—Triiodothyronine, TRH—Thyrotropin-releasing hormone, TSH—Tyroid Stimulator Hormone, TRAb—TSH Receptor Auto Antibodies, hCG—human Chorionic Gonadotropin, D3—type 3 iodothyronine deiodinase.

Thyroid function changes during pregnancy due to involvement of chorionic gonadotrophin in thyroid regulation (Figure 1). Thyroid balance is indispensable for the normal development of the fetus. In the first trimester, hCG acts as a strong stimulator for tyroid stimulator hormone (TSH) receptors, increasing the thyroid function and decreasing the TSH level. TSH and hCG changes mirror each other: when hCG increases, TSH decreases, a condition called gestational thyrotoxicosis [6]. After 12 weeks of gestation, when hCG decreases, TSH increases again. Total thyroxin increases above the upper limit of the reference range for nonpregnant women very early. This is related to the increased liver synthesis of thyroxin-binding globulin, being stimulated by high placental estradiol and therefore, the total thyroxin amount is increased [7].

However, the free thyroxine is kept at the upper limit of reference range for nonpregnant women, and this should be a normal reference for healthy pregnant women. Small amounts of thyroid hormones are passing through placenta at 6 weeks of pregnancy, confirming their important role in embryogenesis [8]. T4 is inactivated by the placenta using type III deiodinase, which converts T4 into rT3, which is biologically inactive. This activity is increasing in the second part of pregnancy.

Maternal TSH is not able to pass through the placenta, but thyrotropin-releasing hormone (TRH) seems to be synthesized in the placenta and contributes to fetal thyroid function, by stimulating fetal pituitary [9].

#### 2.1.2. Hyperthyroidism

Hyperthyroidism in pregnancy has a prevalence of approximately 0.1–0.4% [10]. 

Risks: Untreated or insufficiently controlled hyperthyroidism increases the risk of complications in pregnancy: abortion, premature birth, preeclampsia, retroplacental hematomas, and also maternal heart failure [11].

Uncontrolled severe hyperthyroidism may cause fetal cardiovascular disorders such as tachycardia, cardiomyopathy, cardiac insufficiency, and fetal hydrops [12]. It is also associated with fetal complications: dead at birth, acute respiratory distress, and newborn with low weight according to his gestational age [13]. 

Management: The goal of treatment is to normalize thyroid function with a minimum amount of antithyroid medication. All classes of antithyroid drugs cross the placenta in small amounts, but there is little chance of producing fetal hypothyroidism. Patients should be monitored constantly and the amount of drug administered should be adjusted to maintain free thyroxine (FT4) at the upper limit of the reference range. Women diagnosed with small thyroid goiter can obtain the euthyroid status in two ways: (i) using minimal doses of antithyroid drugs and (ii) discontinuing of medication during the last few weeks of pregnancy [14].

Antithyroid drugs are divided into two categories:

- Thiourea derivative propylthiouracil (PTU) classified by FDA in category D [4], inhibits thyroid peroxidase but also blocks the enzyme type I deiodinase, preventing conversion of T4 to T3, which is more biologically active.
- Imidazole derivatives methimazole (MMI) and carbimazole (CBZ), available in some countries, have the active metabolite methimazole. They inhibit thyroid peroxidase, reducing the synthesis of T4 and T3. Antithyroid drugs are the main pharmacological agents in the treatment of Graves’ disease in pregnant women [15]. Small amounts of PTU, MMI and CBZ cross the placenta and may decrease fetal thyroid function.

Studies have shown that MMI and CBZ classified by FDA category D [4] taken during pregnancy are associated with aplasia cutis (rare disease characterized by growth deficit of skin and hair) and a rare embryopathy consisting of choanal atresia or esophageal and dysmorphic physiognomy [16]; PTU administration in the first trimester was also associated with birth defects, such as urinary tract, face, and neck malformations [17].

Although both drug classes are effective in the treatment of hyperthyroidism, as soon as pregnancy is confirmed the treatment should be adjusted. The current guidelines of Endocrine Society [18] recommend discontinuing previous treatment with MMI/CBZ and change to PTU administration during the first trimester of pregnancy and to switch back to MMI/CBZ for second and third trimesters of pregnancy. This regimen is recommended because MMI is associated with an increased risk of congenital malformations while PTU with maternal hepatotoxicity. 

Doses of these drugs should be adjusted in order to maintain the levels of free-T4 at upper normal values during pregnancy. Serum TSH is primarily used for monitoring thyroid hormone replacement therapy, however, its levels become stable only after four weeks of therapy [19]. Thyroid function in pregnant women with antithyroid medication is recommended to be assessed after 30–40 days from initiation of the treatment and then every 4–6 weeks, according to Endocrine Society guidelines. As pregnancy progresses, drug dose can be reduced, finally discontinuing their medication in the third trimester. In some cases, women with hyperthyroidism may develop anxiety and palpitations. The Endocrine Society’s clinical practice guidelines for management of thyroid dysfunction during pregnancy indicate that the short-term Β-blocker medication propranolol attenuates these symptoms and is not associated with intrauterine fetal growth restriction of the second and third trimesters of pregnancy [18].

If pregnant women with hyperthyroidism have associated asthma and beta-blockers are contraindicated, therapeutic alternatives for treating these symptoms are calcium blockers such as verapamil, although fetal safety data regarding its use during pregnancy are more limited [18]. Resistance to antithyroid treatment during pregnancy is unusual and appears due to reduced therapeutic compliance [20]. Side effects to antithyroid medication may occur in a small percentage of patients (3–5%) [21]. The most common side effect is itching with or without rash, which may occur 2–6 weeks after initiation of treatment. In this case, it is recommended that drug administration be stopped and to change to another antithyroid drug. Other less frequent side effects include migratory polyarthritis, lupus-like syndrome, and cholestatic jaundice [21].

Agranulocytosis is an uncommon side effect and appears very rarely in patients receiving antithyroid medication—only 0.35–0.37% of cases—being determined by a secondary pharmacodynamic mechanism, either toxic or immunoallergic. Agranulocytosis may be asymptomatic, but in some cases clinical symptoms such as fever, chills, and sore throat may occur. It is diagnosed when granulocyte concentration (neutrophils, basophils, and eosinophils) drops below 100 cells/mm³, which represents only 5% of the normal range. Routine monitoring of granulocytes in women with antithyroid medication is not required [22].

Radioiodine I^131^ therapy is contraindicated during pregnancy because it can cause fetal hypothyroidism, when administered after 10 gestational weeks [23]. Therefore in practice, prior to administering a therapeutic dose of I^131^ is mandatory a pregnancy test.

**Figure 2:**
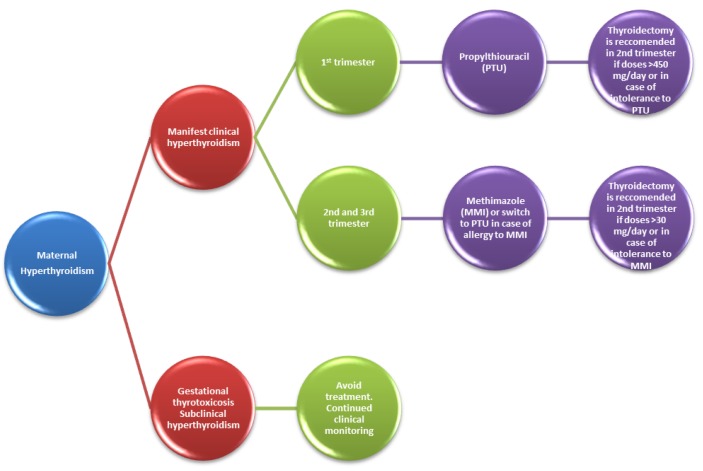
Pharmacotherapeutic management in maternal hyperthyroidism.

Treatment options of hyperthyroidism in pregnancy are summarized in Figure 2.

#### 2.1.3. Hypothyroidism

Primary maternal hypothyroidism is defined as the presence of elevated TSH levels during pregnancy in the absence of other causes such as TSH-secreting pituitary tumors.

##### Manifest Clinical Hypothyroidism

Its prevalence in pregnancy is only 0.3–0.5% [24]. The American Thyroid Association (ATA) defined it as elevated levels of TSH ≥ 10 mU/L regardless of serum-free-T4 levels [25]. The most common cause of hypothyroidism in pregnancy is developing autoimmune chronic thyroiditis (Hashimoto), thyroid surgery history or previous treatment with radioactive iodine for hyperthyroidism, goiter or thyroid cancer.

Risks: Lack of adequate treatment causes maternal complications: premature birth, low birth weight, perinatal death, eclampsia and preeclampsia, anemia, and postpartum hemorrhage [26]. The child with fetal thyroid agenesis is normal at birth, although T4 values are below 1/2–1/3 of normal range [27]. Instead, children from mothers with hypothyroidism show a moderate but significant decrease in intelligence quotient (IQ) [27].

Management: Levothyroxine, classified by the FDA in Group A [4] is the election therapy required in order to achieve a TSH level in the normal range according to gestational age [18,24]. If the pregnant woman has preexisting hypothyroidism and is already receiving L-thyroxine, dosage is determined by gestational age at the time of referral to an endocrinologist:

- Presentation before 12 gestational weeks: patients will require increasing of their levothyroxine dose by 25–50% [18,24]; doses will remain constant after 16 to 20 weeks of gestation until delivery [28,29]. It is recommended to increase the dose of L-thyroxine from 7 therapeutic doses/week to nine therapeutic doses/week, immediately after confirmation of the pregnancy [18,29].
- Presentation after 12 weeks gestation, will evaluate thyroid function tests, and L-thyroxine dosage is based on serum TSH levels [28,29]. Thus:

1. If a TSH level is higher than normal for gestational age—the L-thyroxine dose should be increased;
2. If a TSH level does not exceed the upper normal limit for gestational age—the L-thyroxine dose should not be changed;
3. If the pregnant woman is diagnosed with hypothyroidism and is not being treated with L-thyroxine, thyroid function should be evaluated, and the dosage of L-thyroxine will be chosen based on TSH levels;
4. TSH values >2.5 mIU/mL determine the administration of L-thyroxine in different doses. Therefore, TSH values between 2.5 and 10 mIU/L require 50 L-thyroxine µg daily. For TSH values > 10 mIU/L, the daily dose of L-thyroxine is 100 µg.

**Figure 3:**
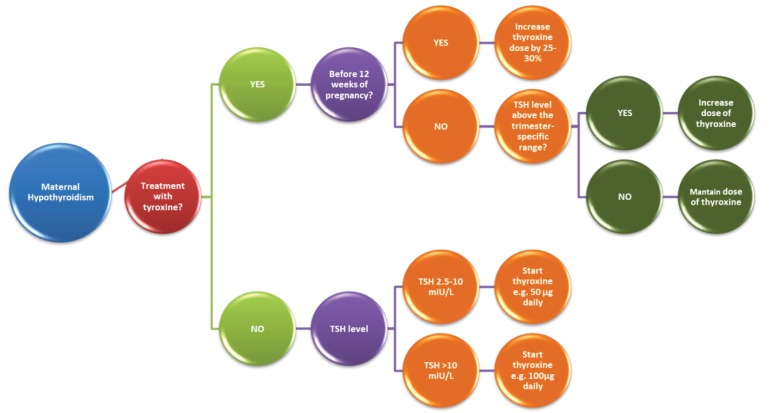
Pharmacotherapeutic management in maternal hypothyroidism.

Treatment options of hypothyroidism in pregnancy are summarized in Figure 3. 

Iodine supplementation is recommended to optimize the normal function of the thyroid gland during the perinatal period. An additional 150 micrograms of iodine daily is recommended to be administered during preconception, pregnancy, and lactation but, without exceeding the limit, excessive iodine >1100 µg per day could determine thyroid dysfunctions [18,24,25,30].

##### Subclinical Hypothyroidism

It has a prevalence of 0.25–2.5% in pregnancies [31], being associated with an increase TSH level (TSH 2.5–10.0 mIU/L) and free thyroxine in normal range [25]. 

Risks: Maternal complications can occur: miscarriages, hypertension, preeclampsia, placental detachment, premature rupture of membranes, neonatal death, and gestational diabetes [32,33,34].

Management: Medical therapy in subclinical hypothyroidism diagnosed in pregnant women is controversial. The American Thyroid Association (ATA) recommends L-thyroxine therapy in women with subclinical hypothyroidism and positive thyroid peroxidase antibody (TPOAb) [24].

The European Thyroid Association (ETA) and The Endocrine Society recommended L-thyroxine to all pregnant women regardless of subclinical hypothyroidism and TPOAb values, until the TSH values reach typical gestational age, considering that the therapeutic benefits are far greater than the disadvantages [35].

Some studies have shown that the number of miscarriages was less in euthyroid pregnant women TPOAb positive and the downward trend in free-T4 and TSH increase with advancing pregnancy [36,37]. 

During therapy for clinical and subclinical hypothyroidism is necessary to perform periodic thyroid function tests, especially in the first half of pregnancy and whenever there is a change in the treatment [35]. Well-controlled hypothyroidism during pregnancy does not justify additional fetal surveillance. In clinical practice, doses of L-thyroxine immediately after birth should be reduced to dosage prior to pregnancy [36,37]. Some guidelines state that these doses are reduced gradually to pre-pregnancy levels within two weeks postpartum, followed by evaluation [31].

#### 2.1.4. Gestational Thyrotoxicosis

Biochemical gestational thyrotoxicosis may be due to increased secretion of hCG, hydatidiform mole, and hyperemesis gravidarum (characterized by severe nausea and vomiting with dehydration, losing 5% of body weight, and ketonuria). This most commonly occurs in multiple or twin pregnancies when serum hCG level is elevated [38,39].

Risks: Pregnancy associated with gestational thyrotoxicosis can lead to miscarriages, birth of dead fetuses, premature birth, preeclampsia, low birth weight, intrauterine growth restriction, and congestive heart failure for the mother if the patient is not receiving treatment [40]. Sometimes, in the absence of a proper treatment, pregnant women with transient gestational thyrotoxicosis may develop severe complications such as thyrotoxic crisis, a real diagnostic challenge [41]. Recent studies have shown variable prevalence of this thyrotoxic crisis: in the US this varies from 0.2% to 0.7% [42], in Europe 2–3% [43], and is much higher in Asia—11% [44]. This is an emergency, consisting of a hypermetabolic condition due to the massive and sudden intoxication with thyroid hormones with severe consequences for mother, especially on the central nervous system (coma), cardiovascular system (shock–collapse) and adrenal glands (acute adrenocortical insufficiency) [45].

Management: Since hCG values decrease after 10 to 12 weeks gestation, antithyroid medication is not necessary, only parenteral hydration and antiemetics [46].

#### 2.1.5. Postpartum Thyroiditis

Postpartum thyroiditis is an inflammation accompanied by thyroid destruction, installed in the first year after birth, and it is a variant of the chronic autoimmune Hashimoto because this is characterized by the presence of antithyroid peroxidase (anti-TPO) antibodies [47,48]. This particular pathology occurs in patients with genetic predisposition and the presence of environmental factors (triggers) [49,50]. Pregnancy is a thyroid trigger postpartum by two mechanisms: cell exchange from mother to fetus [51] and the autoimmune rebound that occurs after birth [52].

Clinical aspects: Generally, the evolution of postpartum thyroiditis is biphasic, beginning with transient hyperthyroidism, followed by hypothyroidism. 

Hyperthyroidism occurs at approximately 1–6 months postpartum, with duration of 2–3 months. Its manifestations are marked by fatigue, irritability, and palpitations; weight loss contrasts with the increased appetite. These are often mild and do not require treatment. In more severe cases, a beta blocker (propranolol or metoprolol) can be used to control the symptoms [53].

Hypothyroidism occurs at 4–8 months postpartum, spontaneously remitting in approximately 4–6 months. Pregnant women with hypothyroidism can be asymptomatic or may accuse myalgia, arthralgia, fatigue, constipation, loss of concentration, weight gain, depression, and may be confused with postpartum depression [54].

Management: The treatment of postpartum thyroiditis is symptomatic. Hyperthyroidism without Basedow’s disease does not require antithyroid medication. In hypothyroidism, substitution with antithyroid medication is required in 30% of cases [55]. Generally, the evolution of the disease is favorable with remittance of symptoms in the majority of cases. In 40% of cases there is a risk of relapse in the next pregnancies [56].

#### 2.1.6. Nodular Thyroid Disease and Pregnancy

Risks factors that lead to the appearance or increase in size of a thyroid nodule during pregnancy are hormone stimulation of the thyroid by hCG and TSH associated with relative immunosuppression and relative iodine deficiency in pregnancy, women who have had two births in the last five years through hormonal stimulation of thyroid in pregnancy and postpartum thyroiditis [57,58]. 

Diagnostic evaluation of a thyroid nodule discovered during pregnancy should be similar to that of nonpregnant patients, the pregnancy having specific problems only regarding the optimal moment of surgical treatment of a nodule suspected as being malignant [59]. Tests of thyroid function are performed in order to detect hypothyroidism or hyperthyroidism. Under uncertain circumstances, evaluation of thyroid autoantibodies or serum calcium (thyroid tumor marker) may be useful. Thyroid radionuclide scanning is contraindicated during pregnancy [60].

Management: Diagnosis and decision-making on general management in the context of a nodular thyroid diagnosed during the pregnancy is primarily based on the results of thyroid ultrasound and fine needle aspiration cytology (FNAC) [60].

**Figure 4:**
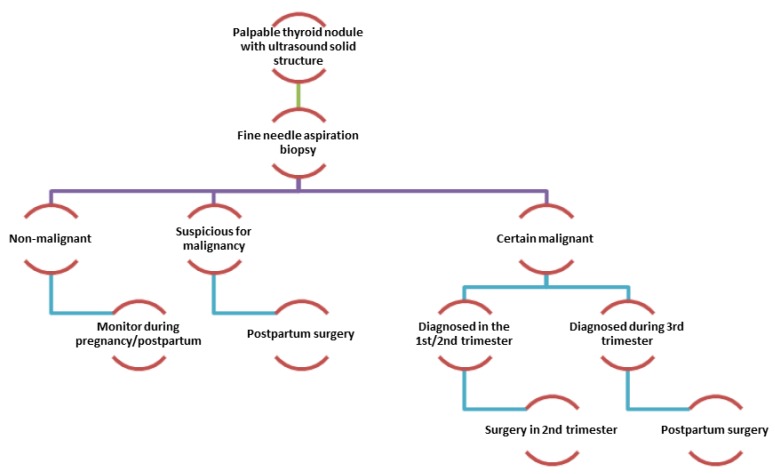
Management algorithm of palpable thyroid nodule during pregnancy.

There are no data to support the need to interrupt the pregnancy if the thyroid nodule that is suspected to be malignant (based on the cytology test or a rapid increase of nodule size) is diagnosed in the first trimester of pregnancy; surgery being indicated in the second trimester (Figure 4). In the case of thyroid nodule diagnosis from the third trimester of pregnancy, the surgery can be delayed after delivery [60].

Studies have shown that postponing of the surgery until after birth, does not change the prognosis: newborn’s birth weight, neonatal morbidity, and mortality or fetal congenital malformations [61]. Treatment with radioactive iodine cannot be given during pregnancy and breastfeeding. A new pregnancy should be postponed one year after radioiodine administration [62].

### 2.2. Parathyroid Disorders and Pregnancy

#### 2.2.1. Calcium Homeostasis in Pregnancy

During pregnancy, calcium homeostasis is maintained by two hormones: parathyroid hormone (PTH) and 1,25-dihydroxyvitamin D (1,25-D). During pregnancy ~30 g of calcium is transferred to the fetus, especially in the last trimester of pregnancy, needed for bone mineralization. The upper limit of normal values for total serum calcium during pregnancy is about 9.5 mg/dL. Lactating women have lower bone density by up to 8% [63]. According to World Health Organization recommendations, from gestational week 20 until the end of pregnancy 1.5–2 g of elemental calcium is necessary daily [64]. 

#### 2.2.2. Hyperparathyroidism

##### Primary Hyperparathyroidism

It presents a very low prevalence in pregnancy of only 0.15%. In the literature, only 200 pregnant women were described with this diagnosis [65]. The first case was described in 1931 [66]. A few years later, the first case of neonatal hypocalcaemia producing tetany in a mother with undiagnosed hypercalcemia due to a hyperparathyroidism was described [67].

Risks: Half of serum calcium circulates bound to plasma proteins, primarily albumin. During pregnancy, due to hypoalbuminemia, increased renal clearance, and placental transfer to the fetus, total serum calcium is slightly decreased. As a result, the parathyroid glands secrete parathyroid hormone in order to maintain calcium homeostasis. This explains why clinical hypercalcemia in pregnant women due to real hyperparathyroidism is diagnosed only in very severe cases, the final confirmation of this diagnosis being postpartum [68]. When serum calcium levels are very high the following disorders can occur: kidney stones, hypertension, cardiac arrhythmias, and pancreatitis.

Management: The only effective treatment for primary hyperparathyroidism diagnosed in the first two trimesters of pregnancy when serum calcium exceeds 11 mg/dL is surgery [69]. For the last trimester diagnosis, treatment strategy is unclear and depends on the severity of hypercalcemia and the clinical condition of the pregnant woman. If surgical treatment was applied, calcium levels should be monitored every 6 h as transient hypocalcemia can occur postsurgery. In this case, calcium should be given intravenously, in the form of calcium gluconate (10%, 10 to 20 mL, for 5–10 min). Such intermittent administration may be repeated or calcium gluconate may be diluted in 5% dextrose or saline isotonic solution (saline) and continuously infused 1 mg/kg body weight/hour [69]. If pregnant women have associated bone disease, postsurgical hypocalcemia can be profound, and requires a more aggressive drug treatment; vitamin D should be supplemented as calcitriol 0.25–0.5 µg/day, classified according to the FDA as category C [4]. 

Drug treatment is reserved for pregnant women who have surgical contraindications and significant hypercalcaemia. It consists in the administration of oral phosphates 1.5–2.5 g/day, but the occurrence of side effects, such as nausea, vomiting, and hypokalemia, requires reducing the drug dose. Calcitonin and cinacalcet are recommended during pregnancy (category C, according to the FDA) and bisphosphonates should be used only when there is no alternative [69]. Other suitable pharmacotherapeutic measures include adequate parenteral hydration, avoidance of drugs which can cause an increase in serum calcium, such as vitamin D, vitamin A, thiazide diuretics, and early treatment of urinary tract infections [69].

##### Secondary Hyperparathyroidism

Secondary hyperparathyroidism is frequently encountered during pregnancy due to increased demand for vitamin D. 

Risks: In the absence of adequate amounts of vitamin D, PTH increases. PTH is not currently tested, only in certain cases as measurement of serum levels of calcium and vitamin D is considered enough for the diagnosis.

Management: As it is frequent in women with poor intake of vitamin D in the diet, malabsorption syndromes, or pigmented or covered skin, supplementation with 25 hydroxy vitamin D (calcidiol) 400–1200 IU per day is sufficient [70]. 

#### 2.2.3. Hypoparathyroidism

Risks: The most frequent cause of hypoparathyroidism in pregnancy is thyroidectomy, and low calcium status can have negative effects on pregnancy and fetal bone development, with increased risk of rickets. It is rarely autoimmune. The first case of hypoparathyroidism in pregnancy was described in 1942, pregnant women may experience cramps, paresthesias, and seizures [71].

Management: Drug treatment is 1,25 hydroxylated vitamin D (alphacalcidol), PTH is necessary for stimulated 1 alpha-hydroxylation. The dose will usually need to be increased during pregnancy because an increased amount of vitamin D is needed [65]. 

### 2.3. Adrenal Disorders and Pregnancy

#### 2.3.1. Physiology of Adrenal Glands in Pregnancy

During pregnancy, the fetoplacental unit produces large amounts of steroid and peptide hormones: CRH (corticotropin releasing hormone) which increases after eight weeks of amenorrhea up to 100 to 1000 times compared with nonpregnancy values; proopiomelanocortin increases after eight gestational weeks, has a maximum concentration at 20 gestational weeks, and then remains stable until birth [72,73]. The plasmatic cortisol doubles its values, mainly through the increase of transport protein under the action of estrogens. The secretion of aldosterone, deoxycorticosterone, and renine is elevated by the secondary aldosteronism [74,75].

#### 2.3.2. Physiopathology of Adrenal Glands in Pregnancy

Adrenal disorders in pregnancy are relatively rare during pregnancy; their pharmacotherapy is important because it is associated with maternal and fetal morbidity and mortality. Diagnosis of these conditions in pregnancy is often difficult, as pregnancy is marked by a series of changes in the endocrine system, including activation of the renin–angiotensin–aldosterone system and the hypothalamic–pituitary–adrenal axis [76].

##### Genetic Disorders: Congenital Adrenal Hyperplasia

Congenital adrenal hyperplasia (CAH) refers to a group of autosomal recessive genetic disorders that arise from failure of steroidogenesis, resulting in the reduced production of cortisol and adrenocorticotropic hormone (ACTH) secondary to increased production [77]. 

Risks*:* There is very little data in the literature about pregnancies in women with CAH.

Management: The goal of drug therapy in CAH in pregnant women is to correct the deficiency of cortisol and suppress ACTH overproduction. The treatment of choice is hydrocortisone 10–15 mg/m^2^/day divided in two or three doses per day, with a higher dose in the evening. Compared to dexamethasone, it is preferred because it is metabolized by the enzyme 11 beta-hydroxysteroid dehydrogenase-2 (11β-HSD2) in placenta and it does not affect the fetus [78]. It is recommended to check 17-OH-progesterone and androgens (testosterone and androstenedione) at least once per trimester. They are increased during pregnancy but normal levels for pregnancy have not been established. Prednisolone, or dexamethasone, which has a longer half-life, may be used if the control is not carried out only with hydrocortisone. They are associated with Cushingoid-like side effects: weight gain and stretch marks [79].

Prednisone is not recommended, since conversion to prednisolone is insufficient used in small doses required for pregnant women with CAH [79]. If mineralocorticoid therapy is necessary, fludrocortisone is administered at 0.05–0.3 mg/day; the dose is adjusted to maintain plasma renin activity at lower levels, no dosage adjustment is necessary for drugs administered in pregnancy.

Dexamethasone treatment in women with CAH starts before the 9^th^ week of pregnancy, before the onset of adrenal androgen secretion and is designed to significantly reduce genital masculinization of women affected by suppression of excessive production of adrenal androgen. Dexamethasone, unlike hydrocortisone, escapes inactivating placental enzyme 11β-HSD2, has a longer half-life, and suppresses the secretion of ACTH. The optimal Dexamethasone dose is 20 µg/kg/day divided in three doses. It is recommended to start treatment as soon as pregnancy is confirmed, and no later than nine weeks after the last menstrual period [80,81].

##### Adrenocortical Hypofunction: Addison’s Disease

The prevalence of primary adrenal insufficiency (Addison’s disease) during pregnancy is very rare—~1:3000 pregnancies—most women being diagnosed before conception [82]. Addison’s disease (AD) is characterized by deficiency of adrenocortical hormones: androgenes, glucocorticoids, and mineralocorticoids. Glucocorticoid and mineralocorticoid deficiency symptoms are nonspecific: weight loss, vomiting, lethargy, and skin hyperpigmentation, which is due to increased ACTH stimulation of melanocytes.

Because the symptoms of pregnancy resemble the clinical suspicion of AD, it must be considered in pregnant women with other associated autoimmune diseases [83]. Besides biochemical pregnancy: hyponatremia, hyperkalemia, increased blood urea and hypoglycemia, low serum cortisol at 9 am, and poor response to synthetic ACTH (Synacthen test). These tests are not as easy to interpret during pregnancy because the increased physiological cortisol levels may lead to “normal” results [83]. 

Risks: Placental unit autonomously produces steroids, and therefore maternal adrenal insufficiency causes no problems in the fetus [83].

Management: The right treatment produces no maternal and fetal complications, especially after the synthesis of cortisone in 1950 [84]. However, there were reports of fetal growth restriction in babies born from mothers with untreated disease [85].

Maintenance treatment in pregnancy includes replacement of glucocorticoid with hydrocortisone and mineralocorticoid with fludrocortisone. Hydrocortisone (category C—FDA) is the treatment of choice for glucocorticoid substitution; unlike other available glucocorticoids, it is degraded by the enzyme 11β-HSD2, it does not cross the placenta, and effects only occur in the mother’s body. The recommended dose is 12–15 mg/m^2^ body surface with 50–75% of the daily dose administered in the morning to mimic the physiological secretion of cortisol [86,87]. Because free cortisol increases gradually with advancing pregnancy, most women with AD require a daily dose of hydrocortisone increased by 20–40%, e.g., 5–10 mg in the third trimester of pregnancy [86,87]. In amniocentesis and caesarean section an initial dose of 100 mg of hydrocortisone is given intravenous (iv) or intramuscular (im) and then, every 6–8 h, the dose is repeated, with gradual reduction in the next 48 h [86]. Doses are increased in women with hyperemesis gravidarum that can be easily mistaken for an adrenal crisis. On the other hand, even hyperemesis can easily trigger an adrenal crisis. 

Treatment of acute adrenal crisis (acute adrenal insufficiency) is a medical emergency and consists in the immediate intravenous bolus administration of 100 mg of hydrocortisone, followed by injection of hydrocortisone 50–100 mg every 6–8 h (or as a continuous infusion of 200–300 mg/24 h) and intravenous saline (originally 1 L per hour, then 200 mL per hour), with regular monitoring of blood pressure, heart rate, and serum electrolytes [86,88].

Fludrocortisone (category C according to FDA) is initiated at a dose of 0.1 mg (dosage ranges from 0.05 to 0.25 mg), and usually the same dose is continued for all the duration of pregnancy. During pregnancy the production of progesterone continuously increases, which exerts antimineralocorticoid activity in vitro and in vivo [89]. Hydrocortisone also acts on the mineralocorticoid receptor and by increasing the dose during the third trimester of pregnancy it can compensate for this; 40 mg hydrocortisone is equivalent to 0.1 mg fludrocortisone [89,90]. To monitor treatment with mineralocorticoid plasma renin may not be used because renin secretion increases physiologically during pregnancy; supine blood pressure is monitored along with serum electrolytes and urinary excretion of sodium [89].

##### Adrenocortical Hyperfunction: Cushing’s Syndrome and Conn Disease

(A) Cushing’s Syndrome

Pregnancy is very rare in women diagnosed with Cushing’s syndrome (CS) due to hypercortisolemia, hyperandrogenemia, and/or hyperprolactinemia that lower fertility [91]. Unlike nonpregnant women, the etiology of CS in pregnancy is represented by benign adrenal tumors produced by the aberrant development of adrenal receptors for LH and hCG [91]. 

Risks: In the absence of adequate treatment in pregnant women with CS maternal and fetal morbidity can occur due to hypertension, preeclampsia, eclampsia, gestational diabetes, congestive heart failure, and pulmonary edema causing miscarriages, stillbirths, and early neonatal deaths.

Management*:* CS treatment in pregnancy should be individualized because improvements or exacerbations of CS may occur; irregular production of placental corticotropin-releasing hormone (CRH) exacerbates hypercortisolemia during pregnancy [92].

If CS is diagnosed in the first trimester of pregnancy, drug therapy should be considered depending on the severity of hypercortisolemia. Tumor removal should be considered from the second trimester [93]. In the case of CS diagnosed in the third trimester of pregnancy, drug treatment is preferred, and surgery should be performed postpartum [92]. The recommended pharmacological agents are the steroidogenesis inhibitors metyrapone, ketoconazole, and mitotane [92,94].

Metyrapone, category C according to FDA [4], is the drug of choice: in 69% of cases it has been shown to reduce hypercortisolemia, but it does not totally control the symptoms [94]. The most troubling adverse effects are determined by increasing the precursors such as 11-deoxycorticosterone, severe hypertension with risk of preeclampsia, intrauterine growth retardation, coarctation of the aorta, and adrenal insufficiency of the newborn [95]. Although animal studies have shown that Metyrapone easily crosses the placental barrier, no human fetal abnormalities were reported [95]. 

Ketoconazole, category C according to the FDA [4], has been used less during pregnancy because of adverse effects: antiandrogenic effect, transient neonatal hypoglycemia, and intense teratogenic effects (only demonstrated in animal studies) [96].

Mitotane (category C—FDA) and other steroidogenesis blockers such as Aminoglutethimide (Category D—FDA) are rarely used and are highly teratogenic, causing fetal masculinization [97]. 

(B) Conn Disease

Risks: Primary aldosteronism (Conn disease), characterized by hypertension and hypokalemia, is extremely rare in pregnancy and should be considered in a pregnant woman with uncontrollable, atypical hypertension, were other causes of preeclampsia were excluded. The association of hypokalemia and hypernatremia with hypertension requires further investigation for adrenal adenoma, adrenal hyperplasia, and adrenal carcinoma [98]. Because during pregnancy urine and plasma aldosterone increase, biochemical test results must be correlated with the normal values for pregnancy.

Management: Drug treatment of primary aldosteronism is limited because in pregnancy are contraindicated aldosterone antagonists such as Spironolactone—a potassium-sparing diuretic (category C—FDA) [4]—angiotensin converting enzyme inhibitors, and angiotensin II receptor antagonists. Also, Spironolactone has antiandrogenic effects and feminizes the male fetus [90].

Eplerenone, mineralocorticoid receptor antagonist (category B—FDA), has been reported to be successfully used in a single case [90], the same as Amiloride, a potassium-sparing diuretic [99]. Potassium salts can be used when blood pressure is controlled by methyldopa, nifedipine, or labetalol.

##### Adrenomedullary Hyperfunction: Pheochromocytoma 

Pheochromocytoma is a tumor of the adrenal medulla (90%) but could also be localized in other areas of the sympathetic nervous system. 

Risks: It produces adrenaline and noradrenaline and is a secondary cause of high blood pressure in pregnancy [100]. The prevalence in pregnancy is very low (0.007%) and can be fatal for the pregnant woman and the fetus because of extreme paroxysms of blood pressure, often imitating preeclampsia, but with much higher fetal mortality [100]. Typical symptoms include palpitations, excessive sweating, labile blood pressure, causing orthostastic hypotension, and significant hypertension. There are increased catecholamines in the urine and specific metabolite vanilmandelic acid, due to the excessive sporadic release of catecholamines from the tumor, which may be located in the adrenal medulla or anywhere along the sympathetic chain [101].

Management: The goal of drug treatment is to normalize blood pressure and pulse. Among α-adrenergic-blocking pharmacological agents the most often used is phenoxybenzamine (category C according to FDA), which is safe in pregnancy and can be initiated at doses of 10 mg/day; the dose may be increased to 10 mg every 2 to 3 days until symptoms and blood pressure are controlled [102]. Subsequently, dosage adjustment may be necessary because phenoxybenzamine has a half-life of ~24 h and therefore the effect is cumulative. The usual dose is 40–80 mg/day in divided doses. The maximum dose of phenoxybenzamine is 1 mg/kg/day [102].

Other selective alpha1-antagonists for use in pregnancy are Prazosin (category C—FDA) and Doxazosin (Category C—FDA); their α-adrenergic receptor blocking activity increases blood volume and can improve congestive heart failure and angina pectoris, if they are combined; it is recommended to administer them for 7 to 14 days if surgical treatment is indicated [102,103].

Beta-blockers such as propranolol (category C—FDA) are recommended only after taking alpha blockers. Hypertensive crises can be treated with intravenous phentolamine (category C—FDA).

### 2.4. Pituitary Disorders and Pregnancy

#### 2.4.1. Pituitary’s Physiology in Pregnancy

All hormonal axes of the body are directed by the pituitary gland; most are activated and operational during pregnancy except for the ovarian hormone axis, which is inhibited due to high serum levels of estrogens and progesterone [104]. In pregnancy the secretion of prolactin from the anterior pituitary lactotroph cells increases;conversely, the pituitary gland grows in size up to two or three times. As opposed to lactotroph cells, the rest of the cells in the anterior compartment of the pituitary remain at pre-pregnancy size or even smaller [104]. Starting up with 16–17 weeks of pregnancy, the placenta secretes the placental growth hormone which in turn leads to increase of insulin-like growth factor (IGF). IGF inhibits pituitary somatotropic cells and the secretion of somatotropic hormone (STH) decreases [105]. The corticotropic axis is also modified because placenta secretes corticotropin releasing hormone (CRH) [105]. TSH (thyrotropic hormone) plasma levels decrease in parallel with the increase in hCG (human chorionic β-gonadotropin) levels, which has also affinity for the TSH receptor [106]. Even if the vasopressin concentration remains stable, in pregnancy it decreases the osmotic threshold of thirst and plasma arginine vasopressin secretion, consequently, the plasma osmolarity and natremia decrease [106].

#### 2.4.2. Pityitary’s Physiopathology in Pregnancy

Pituitary disorders are uncommon in pregnancy, some occur during or after pregnancy, such as Sheehan’s syndrome (postpartum hypopituitarism), others are preexisting to the pregnancy, such as prolactinomas, Cushing’s disease, and acromegaly. Pituitary insufficiency prevalence is very low, as there are only about 42 diagnosed cases/million [107].

##### Tumoral Pathology of Pituitary and Pregnancy: Prolactinoma

Prolactinoma is the most common cause of persistent hyperprolactinemia and represents 50% of pituitary tumors [108]. These are predominantly benign tumors and classified as microprolactinomas (size < 10 mm) or macroprolactinomas (size ≥10 mm).

Risks: 

Effect of prolactinoma on pregnancy. Hyperprolactinemia has no effect on pregnancy or fetal development, but there is a risk of estradiol receptor-related tumor expansion [109].

Effect of pregnancy on prolactinoma*:* During pregnancy, the pituitary undergoes global hyperplasia due to increased serum levels of estrogenes leading to tumor growth and the possibility of visual loss [108]. Microadenomas of less than 10 mm rarely cause problems during pregnancy, while macroprolactinomas larger than 10 mm require increased attention regarding pharmacotherapeutic management [110]. Women with macroprolactinomas require visual field testing every trimester and in case of visual impairment treatment with dopamine agonists (DA) is initiated [111,112]. When there is a risk of optic chiasm compression DA are recommended throughout pregnancy.

Management during pregnancy*:* The objective of pharmacological treatment depends on the size of the adenoma, the target being maintaining the prolactinoma away from the optic chiasm and levels of prolactin to be within the normal range for pregnancy [110]. Lack of efficacy of drug therapy requires transsphenoidal surgery for removal of adenomas, only in the second trimester [111].

Prolactin secretion is under negative feedback control by dopamine, and consequently DA like Bromocriptine and, more recently, Cabergoline, is the mainstay of treatment in nonpregnant as well as pregnant women. 

Bromocriptine, category B according to the FDA [4], is the DA of choice for pharmacologic treatment of prolactinoma during pregnancy, some studies confirming no adverse effects in treating over 6000 cases [113]. The effective dosage is 2.5–5 mg/day, seldom being required at 7.5 mg/day or more. Bromocriptine is effective not only for the normalization of prolactin level, but also for reducing tumor size [113].

Cabergoline, category B according to FDA [4], is the alternative DA, because it has a good safety profile without teratogenic effect; although it is not recommended for prolactinoma pharmacotherapy in pregnancy because long-term use (over 1 year) has been associated with fibrosis of the heart valves and constant supervision by echocardiography is necessary [114].

Management after pregnancy: After birth, microadenoma presents a negligible risk and breastfeeding is permitted. In case of macroadenoma, breastfeeding is not recommended. It is also recommended to control prolactin and pituitary morphology three months after birth [115]. Sometimes it was found a total remission of a microprolactinoma (probably by vascular effect) after birth [116].

##### Hyperactivity of Pituitary Gland and Pregnancy: Acromegaly

The occurrence of pregnancy in women with acromegaly is very rare, because the hypersecretion of growth hormone from the pituitary somatotrophs leads to lack of ovulation. The simultaneous appearance of hyperprolactinemia in 40% of cases, with or without macroadenoma, also affects fertility [117]. The first case of normal pregnancy in a woman with acromegaly was reported in 1954 [93].

Risks: Comorbidities in pregnant women are numerous: hypertension, preeclampsia, gestational diabetes due to insulin resistance resulting from the anti-insulin effect of growth hormone (GH), and heart disease [60,77]. There is also a potential risk of tumor expansion due to growth stimulation by estrogen, which leads to neurological complications and/or visual complications due to proximity to the optic chiasm, and transsphenoidal surgery is recommended [117].

Management: Dopamine agonists can control pregnancy acromegaly in 10% of cases [118]. Bromocriptine (category B—FDA) is considered safe and is more efficient when there are cosecretant GH and prolactin (PRL) tumors. A higher prevalence of microsomic fetuses in pregnant women treated with bromocriptine was found however compared to untreated women [118]. Somatostatin analogs, octreotide (category B—FDA), or lanreotide (category C—FDA) cross the placental barrier;their safety in pregnancy has not been precisely established [119]. All five subtypes of receptors and somatostatin, including somatostatin receptor type 4 (SST4) which has a reduced affinity for Octreotide, are present in the placenta and umbilical cord, suggesting that the maternal–fetal barrier carries a weak functional response to somatostatin analoganalogs [119]. For the treatment of acromegaly, Octreotide is also administered before pregnancy, as a result, the drug is administered in the first few weeks after conception, but no fetal malformation was reported [119].

Studies have demonstrated that administration of somatostatin analoganalogs in pregnant women with acromegaly has been correlated with intrauterine fetal growth retardation and birth of children with low weight (growth-restricted), compared to those who did not receive them [119]. A partial explanation would be the transient reduction of the uterine artery flow and of systolic velocity after the administration of short-acting Octreotide [120]. Pegvisomant (category B—FDA) has proven to be a safe and effective drug; fetal concentration is minimal suggesting a low or absent transplacenta passage [120,121]. Although medical treatment of pregnant women with acromegaly is not associated with major side effects of the mother or fetus, it should be discontinued during pregnancy. If medical treatment continues, close monitoring of fetal development is recommended.

##### Hypoactivity of Pituitary Gland and Pregnancy

(A) Hypopituitarism during Pregnancy

Risks: Pregnant women with hypopituitarism (deficiency of hormones secreted by the anterior pituitary), usually secondary to pituitary tumors, have a high risk of cerebrovascular disease and, therefore, pharmacotherapy is very important during pregnancy [122].

Management: Glucocorticoid of choice in pregnant women with ACTH deficiency is hydrocortisone 20–30 mg/day (two-thirds of the total dose in the morning and a third in the evening), (category C—FDA). In some clinical situations, the amount of hydrocortisone may be reduced by one third of the total dose, as the effects of cortisone are boosted by estrogen during pregnancy due to increasing in corticosteroid-binding globulin [123]. The therapeutic alternative to hydrocortisone is represented by synthetic corticosteroids: 5.0–7.5 mg prednisone daily and dexamethasone 0.5–0.75 mg per day, (category C—FDA), mentioning that they are not boosted by estradiol. Because these pregnant women have ACTH deficiency, aldosterone secretion is normal and there is no need of mineralocorticoid replacement therapy.

For Levothyroxine (category A—FDA), the usual dose of 0.1–0.2 mg is adjusted so free-T4 levels are included in the upper normal range for gestational age [55] because small values of free-T4 especially in the first quarter have a negative impact on psychomotor child development [124]. TSH is not a useful marker to monitor treatment with Levothyroxine during pregnancy.

Genetic recombinant GH treatment is not approved for administration during pregnancy, although some studies have shown women who became pregnant during treatment with GH and it has no longer been administered in the first trimester had pregnancies with good evolution and development of normal children [125]. Desmopressin (category B—FDA) administration is considered to be safe during pregnancy, nonteratogenic. Although it has similar chemical structure to vasopressin, it does not seem to increase the frequency or amplitude of uterine contractions although it has oxytocin-like properties [126].

(B) Postpartum Hypopituitarism: Sheehan Syndrome

Sheehan’s syndrome (SS) is a very rare condition resulting from infarction and necrosis of the pituitary gland [127]. 

Risks: The pituitary gland, increased physiologically during pregnancy, causes upper pituitary artery compression and hypotension secondary to severe bleeding during childbirth and postpartum causes small blood vessel spasm, apoplexy and subsequent necrosis of the anterior pituitary. 

Management: Hormone replacement therapy is fundamental in SS. Glucocorticoid administration is done before treatment with L-thyroxine [127]. 

(C) Panhypopituitarism during Pregnancy

Panhypopituitarism is characterized by a global deficit in anterior and posterior pituitary hormones. A pregnancy in these cases is rare, with an unpredictable evolution as there are only a few reports of cases in the literature. Thus, Shinar et al. recently reported four cases of pregnant women, aged between 26 and 31 years, diagnosed with panhypopituitarism without any detectable pituitary function modification in repeated tests. They delivered to term with spontaneous labors. The pregnancies were obtained using in vitro fertilization and these have been treated all the pregnancy period with hormonal replacement therapy: glucocorticoids, vasopressin, and thyroxin. In none of the four cases the breastfeeding was possible due to the lack of prolactine [128].

Tonda et al. presented the case of a young pregnant woman with severe clinical disturbances, such as visual disturbances, intense headache, insipid diabetes, polydipsia, and polyuria, which gave birth prematurely (gestational age 32 weeks). Blood tests revealed panhypopituitarism and imaging explorations showed a pituitary lesion. After six weeks of adequate hormone replacement therapy the imaging explorations showed a normal pituitary gland [129].

##### Diabetes Insipidus

Diabetes insipidus (DI) is a rare disorder characterized by antidiuretic hormone (ADH) deficiency. Produced in the hypothalamus and stored in the posterior pituitary, ADH acts directly on the distal tubules to allow water reabsorption in response to dehydration [121]. DI can be a result of ADH hyposecretion in the posterior pituitary gland (central DI, endocrine) or lack of response to its action in the kidney (nephrogenic DI, peripheral) [121]. As a result, there is an inability to maintain normal plasma osmolarity (275–295 mmol/L) and urine concentrations (>300 mOsmol/kg). Polyuria (>3 lit/24 h) stimulates increased thirst with compensatory polydipsia [121]. Plasma osmolality decreases by 5 to 10 mOsm/kg shortly after conception and throughout pregnancy remains low [130]. Accordingly, ADH level remains low throughout pregnancy, especially in the first and second trimester of pregnancy [131]. The ability to concentrate urine remains in the normal range [132]. The prevalence of DI during pregnancy is low, ~1:30,000 [133]. 

Transitional DI may occur in the last trimester of pregnancy, due to increased glomerular filtration rate, renal prostaglandins increase with ADH antagonism, and placental production of vasopressinase, an ADH degrading enzyme [134]. 

Risks: Morbidities may occur due to preeclampsia, acute fatty liver of pregnancy, and HELLP syndrome (hemolysis, elevated liver enzyme levels, and low platelet levels). The lack of therapeutic effect of vasopressin is due to the significant increase in plasma clearance of antidiuretic hormone (ADH) [134]. This explains also the good response to desamino-D arginine vasopressin (1-desamino-8-D-arginine vasopressin) DDAVP (Desmopressin, Minirin) [135], which is not metabolized by vasopresinase.

Management: DI pharmacotherapy in pregnancy is approached differently depending on etiology.

(A) Central and Transient Diabetes Insipidus

At the beginning of the eighteenth century, drug therapy in pregnancy DI was aggressive hydration and increased fluid intake. At the beginning of the nineteenth century posterior pituitary extract was first used in the treatment of pregnancy DI [136]. The extract has an irritating and sensitivity effect on the nasal mucosa and it induces formation of antibodies. Since this bovine or porcine extract contains oxytocin in addition to vasopressin, undesirable complications have occurred [136,137]. New drugs have been synthesized containing exogenous vasopressin: pitressin—an aqueous solution of vasopressin (vassopresin 20 units/mL); pitressin-tannate—oily solution (vasopressin 5 units/mL); and lypressin (lysin-8-vasopressin)—nasal spray (vasopressin 2 units per spray) but even they are rapidly catabolized by placental vasopressinase due to chemical structures similar to endogenous vasopressin [138]. Therefore, in women with DI receiving this medication, disease control was not achieved because as the pregnancy advances and placental vasopressinase level increased.

Currently the elective drug treatment for central, preexisting DI and transitional pregnancy DI is 1-deamino-8-D-arginine-vasopressin (DDAVP) (desmopressin, minirin), category B—FDA. DDAVP is a selective activator of V2 receptors in the distal nephron inducing increased water retention with the emergence of hyponatremia [139]. DDAVP is an analog of vasopressin with a modified N-terminus and a change in the chemical structure of arginine at position 8, and is therefore resistant to the catabolic action of placental vasopressinase [140]. 

If central preexisting DI was controlled by DDAVP before pregnancy, treatment may be continued during pregnancy, sometimes requiring an increase in dose. Diagnosis of DI during pregnancy requires the same drug therapy to that outside of pregnancy [141]. DDAVP can be administered intranasally, subcutaneously, intravenously, or orally in a starting dose of 10 mcg intranasally before sleep to prevent nycturia. The intranasal or sublingual route is recommended during pregnancy, rarely parenteral [142]. Higher doses can sometimes be necessary: the effective dose depends on the severity of DI (partially or totally deficient vasopressin) and individual parameters, such as absorption, which affect the pharmacokinetics and pharmacodynamics of the drug. The therapeutic effect is long [141,142]. DDAVP standard doses are between 1 and 2 µg once or twice daily by injection; 5 to 20 micrograms twice or three times a day, nasally; and 60 to 120 µg two or three times a day, via the sublingual route [141,142]. 

For nasal administration, either a graduated tube is used (allowing a variable amount of product to be administered) or a nasal spray, containing about 10 µg per puff of the drug. Sublingual administration is simple, using desmopressin lyophilized and dissolved under the tongue [135]. Posology is adjusted to the symptoms: the therapeutic target is the elimination of polyurodipsic syndrome and the avoidance of drug overdose [141,142]. Usually the pregnant woman shall establish the optimal dose herself, increasing the evening dose gradually until she no longer drinks water at night and nycturia disappears, and then is advised to use the same procedure during the day.

The main side effect of DDAVP is water intoxication due to overdose, the volume of water ingested is disproportionate to the removed volume because desmopressin blocks diuresis and causes hemodilution. This results in hyponatremia, oliguria, edema, headache, lethargy, nausea and vomiting [135]. Seizures and coma may occur due to hyponatremia. In order to avoid the risk of water intoxication, the patient should be advised to drink only when thirsty. It is also recommended to omit a dose of DDAVP once a week, for example. By producing transient polyuria any gradually accumulated fluid retention is eliminated, thus correcting fluid balance. The pregnant woman may then resume the usual dose of desmopressin [143]. Monitoring urine output, fluid intake restriction and monitoring of serum sodium osmolality and urine osmolality can minimize the risk of osmotic complications secondary to DDAVP administration in pregnant women with DI [135].

In transient pregnancy DI, DDAVP can usually be stopped a few days or weeks before birth because postpartum, vasopressinase produced by the placenta disappears [143]. Although classified by the FDA as risk category B [4], there continues to be a lack of controlled studies for DDAVP to provide data on its safety for mother and fetus [126]. The great advantage of DDAVP is that being a selective V2 receptor activator, it avoids stimulating uterine contractions or blood pressure compared with exogenous vasopressin forms [126].

(B) Nephrogenic Diabetes Insipidus

Management: Congenital nephrogenic DI therapy in pregnant women is very rare and requires the corrections of the abnormalities of calcium or potassium. Hydrochlorothiazide is used conventionally to treat nephrogenic DI [144]. Although included in category B by the FDA, the pregnant women need precautions to avoid hypovolemia and hyponatremia in mother and fetus. Hydrochlorothiazide can create difficult problems during labor due to the difficulty of balancing abundant polyuria by an equivalent fluid intake [144]. 

## 3. Conclusions

Pharmacotherapeutic management of endocrine diseases associated with pregnancy is complex and covers fetal, neonatal, and maternal issues that may occur during gestational period. Women with various antenatal diagnosed endocrine diseases should be counseled about the possible complications that may occur along the way. Medication and monitoring should be made by a multidisciplinary team; excessive doses should be avoided.

The management is much more difficult if endocrine disorders are discovered during pregnancy; the symptoms are sometimes indistinguishable from those that occur physiologically during pregnancy. Thyroid disorders are the most common endocrine problems associated with pregnancy, but subclinical thyroid disease treatments generate controversy; the therapeutic target in these cases should be to maintain euthyroidism throughout pregnancy.

The most common of pituitary tumors are microprolactinomas. If they have macroprolactinomas, pregnant women should be tested constantly for visual field. In both cases it is important that patients be counseled about the occurrence of intense headache requiring emergency medical presentation as tumor expansion may compromise the optic chiasm.

Clinical suspicion for Cushing’s syndrome, pheochromocytoma, and Conn’s syndrome should be considered in women with high blood pressure who do not respond to classic, conventional treatment during pregnancy.
